# Achilles Subtendon Structure and Behavior as Evidenced From Tendon Imaging and Computational Modeling

**DOI:** 10.3389/fspor.2020.00070

**Published:** 2020-06-23

**Authors:** Geoffrey G. Handsfield, Joachim Greiner, Josef Madl, Eva A. Rog-Zielinska, Enzo Hollville, Benedicte Vanwanseele, Vickie Shim

**Affiliations:** ^1^Auckland Bioengineering Institute, University of Auckland, Auckland, New Zealand; ^2^Institute for Experimental Cardiovascular Medicine, University Heart Center Freiburg Bad Krozingen, Bad Krozingen, Germany; ^3^Faculty of Medicine, University of Freiburg, Freiburg, Germany; ^4^Human Movement Biomechanics Research Group, Department of Movement Sciences, KU Leuven, Leuven, Belgium

**Keywords:** finite element, MRI, second harmonic, biomechanics, function

## Abstract

The Achilles tendon is the largest and strongest tendon in the human body and is essential for storing elastic energy and positioning the foot for walking and running. Recent research into Achilles tendon anatomy and mechanics has revealed the importance of the Achilles subtendons, which are unique and semi-independent structures arising from each of the three muscular heads of the triceps surae. Of particular importance is the ability for the subtendons to slide, the role that this has in healthy tendons, and the alteration of this property in aging and disease. In this work, we discuss technical approaches that have led to the current understanding of Achilles subtendons, particularly imaging and computational modeling. We introduce a 3D geometrical model of the Achilles subtendons, built from dual-echo UTE MRI. We revisit and discuss computational models of Achilles subtendon twisting suggesting that optimal twist reduces both rupture loads and stress concentrations by distributing stresses. Second harmonic generation imaging shows collagenous subtendons within a rabbit Achilles tendon; a clear absence of signal between the subtendons indicates an inter-subtendon region on the order of 30 μm in our rabbit animal model. Entry of wheat germ agglutinin in both the inter-fascicular and the inter-subtendon regions suggests a glycoprotein-containing inter-subtendon matrix which may facilitate low friction sliding of the subtendons in healthy mammals. Lastly, we present a new computational model coupled with human exercise trials to demonstrate the magnitude of Achilles subtendon sliding which occurs during rehabilitation exercises for Achilles tendinopathy, and shows that specific exercise can maximize subtendon sliding and interface strains, without maximizing subtendon strains. This work demonstrates the value of imaging and computational modeling for probing tendon structure-function relationships and may serve to inform and develop treatments for Achilles tendinopathy.

## Introduction

In the last decade, research has expanded rapidly in the areas of Achilles tendon structure and mechanics. One particularly exciting research direction pertains to the Achilles subtendons, the presence and behavior of which have only recently been reported (Szaro et al., 2009; Handsfield et al., 2016). Achilles subtendons are separate and semi-independent regions of the Achilles tendon, each arising from a different muscular head of the triceps surae—soleus, medial gastrocnemius (MG), and lateral gastrocnemius (LG) (Handsfield et al., 2016). Functionally, each subtendon is similar to a unique tendon as it transmits force from a single muscle belly. Yet each is part of the larger superstructure of the Achilles tendon and does not have fully independent motion and function. The earliest identification of the subtendons as semi-independent structures was reported by Szaro et al. (2009) when that group conducted careful dissection of human cadaveric Achilles tendons, but dissected from muscle belly toward the calcaneus. In doing so, Szaro et al. recognized and documented the presence of unique independent structures of the Achilles tendon, each associated with one of the heads of the triceps surae, and this work was subsequently replicated (Edama et al., 2014, 2016). The importance of this work may have been underappreciated as these groups reported the structures as fascicles of the Achilles tendon, and they were thus not recognized as previously unidentified structures. Handsfield et al. (2016) argued that the structures these groups had probed were on a larger size scale than fascicles and must represent a previously unreported meso-scale structure. Subtendons are composed of many fascicles and represent a functional portion of the whole Achilles tendon (Handsfield et al., 2016).

Since the recognition and reporting of subtendons, there has been a surge in tendon and subtendon research, including imaging (Handsfield et al., 2017; Clark and Franz, 2020), computational modeling (Handsfield et al., 2017; Shim et al., 2018), anatomical dissection (Pekala et al., 2017; Mahan et al., 2020), and experimental approaches (Finni et al., 2018; Maas et al., 2020). Structurally, the subtendons display an internal torsion or twisting, which can be seen by the naked eye by observing the trajectory of collagen fascicles within the Achilles (White, 1943; van Gils et al., 1996). Functionally, subtendon sliding has been proposed, as subtendons are believed to slide past one another semi-independently, similar to what has been demonstrated for fascicles (Thorpe et al., 2013, 2015). This potential mechanism offered an explanation to non-intuitive motion observed *in vivo* in the human Achilles that had previously been unexplained (Arndt et al., 2012; Slane and Thelen, 2014; Franz et al., 2015). Like the sliding that occurs between fascicles of energy-storing tendons, sliding between subtendons may be a normal property of healthy Achilles tendons that may be diminished in aging or tendinopathic tendons (Slane and Thelen, 2014; Clark and Franz, 2020). Sliding of Achilles subtendons has now been demonstrated in computational models and animal experiments (Handsfield et al., 2017; Finni et al., 2018; Maas et al., 2020), and is consistent with *in vivo* observations of dynamic movements using ultrasound imaging (Slane and Thelen, 2014; Franz et al., 2015). The nature of the interface between the subtendons is still unknown and this critically limits our understanding of the mechanics of inter-subtendon sliding.

Considerable progress in the field of tendon biomechanics has come from advances in imaging and computational modeling. *In vivo* imaging methods are a powerful tool for studying human tendon as they allow for direct investigations into the anatomy of a living patient or volunteer. Anatomy can then be assessed, compared to functional measurements of that individual, or used to build subject-specific or generalized computational models. Ultrashort echo time (UTE) MRI, for instance, allows for in-depth human tendon modeling and subject-specific analysis of tendon structure and mechanics. Imaging of animal models with second harmonic generation (SHG) imaging (Campagnola and Loew, 2003) allows a deeper probing into the micro-scale structure of the tendon. Computational modeling can explore tendon mechanics based on imaging-derived geometry and creates the opportunity for *in silico* experimentation where features and boundary conditions can be added, removed, or altered in order to explore the effects on tendon mechanics and behavior in ways that are impractical or impossible for experimental approaches. For example, *in silico* experiments can be used to explore the effect of diverse structural features or boundary conditions on the muscle-tendon system (Handsfield et al., 2017; Shim et al., 2018). Much more is still to be learned about subtendon behavior in humans, and computational modeling is an avenue for exploring subtendon motion during the activities of daily living, where force and motion data can be experimentally measured and subtendon mechanics can be modeled and probed.

In this work, we aim to reexamine our understanding of Achilles subtendon structure and mechanics, particularly that which we have explored with imaging and computational modeling. We discuss UTE MRI in its contribution to Achilles tendon imaging and present an optimized method for obtaining high-signal high-contrast images of the tendon. We perform SHG imaging in animal models to provide preliminary evidence for the existence of an inter-subtendon matrix in the Achilles tendon, defined by an absence of fibrillary collagen. Lastly, we introduce a new computational finite element model that predicts the motion of subtendons and the amount of subtendon sliding based on experimentally recorded muscle forces and joint motions during rehabilitation exercises for tendinopathy, which is discussed in the context of previous computational models revealing the mechanisms of fascicle twist and subtendon twist and sliding in the Achilles tendon.

## Materials and Methods

### Ultrashort Echo Time MRI

**Figure 1:**
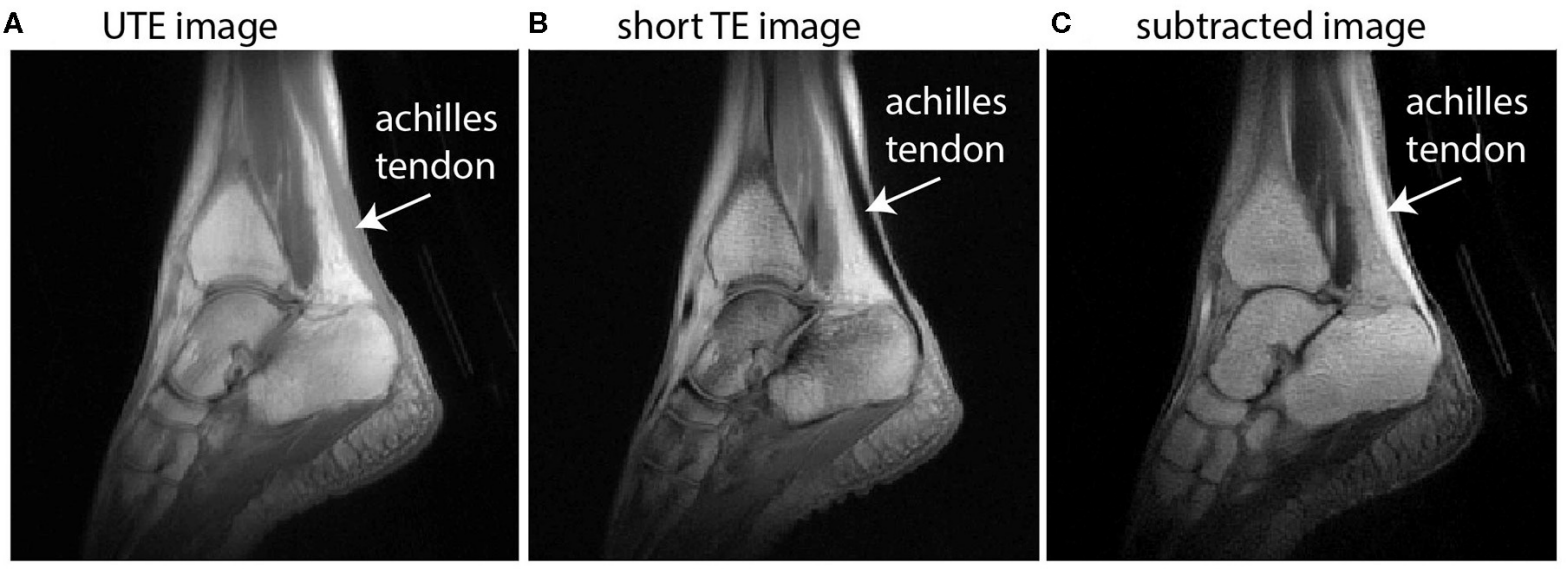
Sagittal images of the ankle illustrate the principle of dual-echo UTE imaging of the tendon. UTE images **(A)** implement a TE that is short enough to acquire signal in the Achilles tendon but with low signal and low contrast between other tissues, particularly muscle. A conventional proton-density weighted image with short TE **(B)** is absent of any signal from the Achilles tendon. The subtracted image **(C)** offers high signal in the Achilles tendon and high contrast with neighboring tissues.

Imaging collagen-rich tissues with very short T2^*^ decay properties, such as tendons, is a challenge for conventional MRI (Fullerton et al., 1985) since signal cannot generally be obtained from tendon or other collagen-rich tissues when echo times (TE) exceed ~1 ms (see Figure 1B). UTE MRI uses non-Cartesian k-space trajectories to acquire images at ultrashort TEs (e.g., TE <0.1 ms). UTE imaging has been achieved in the past using stacks of spirals (Qian and Boada, 2008; Qian et al., 2012) or 3D radial spoke acquisitions (Miller et al., 2015). For Achilles tendon MRI, UTE methods need to (i) acquire signal in the Achilles tendon and (ii) achieve contrast between the Achilles tendon and its neighboring tissues. Neighboring tissues may be subcutaneous fat, retrocalcaneal bursa, cortical bone, free fluid in the ankle, or adjacent muscles, e.g., the soleus or flexor hallucis longus.

In this work, we use dual-echo UTE imaging and image subtraction—where two images taken at the same location are subtracted to change the signal characteristics—to overcome these challenges and achieve *in vivo* images with high signal throughout the Achilles tendon and high contrast between the Achilles and its neighboring tissues (see Figure 1). Briefly, dual-echo UTE MRI acquires signal at two echo times—an ultrashort TE (here: 0.08 ms) and a short TE (here: 2.54 ms). Since image subtraction is implemented, the second echo time should be long enough that nearly all of the tendon signal has decayed away, but short enough that the muscle signal is similar between the two echoes. With increasingly long second TEs, image subtraction produces higher signal in the muscle tissue, which diminishes contrast between tendon and muscle. UTE images were acquired from a healthy female volunteer (height/mass/age: 163 cm/52 kg/27 years) on a 3T Siemens Trio MRI Scanner. Imaging sequence details have been described previously (Handsfield et al., 2017) and the non-Cartesian acquisition method is based on Miller et al. (2015). Here, we report an optimized sequence for dual-echo UTE and present new images to illustrate the methodological advantages of using dual-echo UTE for tendon imaging. Briefly, our sequence used the parameters TE1(UTE)/TE2(shTE)/TR/α: 0.08 ms/2.54 ms/6 ms/10°. In plane spatial resolution was 0.8 mm × 0.8 mm × 0.8 mm. Short TE images were subtracted from UTE images to maximize tendon signal and contrast (Figure 1).

### Modeling Tendon and Subtendon Geometry From Dual-echo UTE

The creation of a 3D geometrical model of the Achilles subtendons entailed, first: segmenting the Achilles tendon in subtracted dual-echo UTE images (see Figure 1C). Segmentations were used to reconstruct a 3D model of the Achilles tendon in Autodesk Inventor software (Autodesk, San Rafael, CA, USA). Note that our imaging routine did not enable us to resolve individual subtendons, so our initial 3D model was of the whole Achilles. To explore subtendons within the 3D tendon, anatomical literature was used to inform the location of the three subtendons of the Achilles at the inferior/distal end of the free tendon (Sarrafian, 1993; Szaro et al., 2009). At the superior/proximal end of the free tendon, the locations of the subtendons were determined based on the anatomical location of the associated muscle. For example, at the proximal free tendon, the lateral gastrocnemius (LG) subtendon was modeled as the lateral posterior aspect of the tendon. Cutting planes were created in Autodesk Inventor that twisted 90° and subdivided the Achilles subtendons continuously from the proximal to the distal end of the free tendon. The calcaneal insertion was defined as the region of the tendon that was adjacent to the calcaneus. Cutting planes were made to extend through the calcaneal insertion region without twisting, as this is the region where subtendons fuse with the calcaneus. The cutting planes resulted in three subtendons that were consistent with literature descriptions of subtendons (Edama et al., 2014, 2016).

### Second Harmonic Generation (SHG) Imaging

Second harmonic generation (SHG) imaging is a label-free microscopy method that utilizes a non-linear optical effect to visualize certain molecular structures, such as collagen fibers (Campagnola and Loew, 2003; Kahn et al., 2013). It can be combined with two-photon fluorescence microscopy which then allows visualizing of non-collagenous structures. All investigations reported in this study conformed to the German animal welfare laws (TierSchG and TierSchVersV), compatible with the guidelines stated in Directive 2010/63/EU of the European Parliament on the protection of animals used for scientific purposes, and they were approved by the local Institutional Animal Care and Use Committees in Germany (Regierungspräsidium Freiburg, X-16/10R). Animal housing and handling was conducted in accordance with good animal practice, as defined by the Federation of European Laboratory Animal Science Association, FELASA. The Achilles tendons from one 10-months-old female New Zealand white rabbit were dissected after sacrifice by injection with sodium pentobarbital solution. Rabbits are a convenient model as they are among the largest of animals that can be housed in small animal suites, their Achilles tendons are relatively large and elastic, and their Achilles is composed of distinguishable subtendons (Doherty et al., 2006). One tendon was immediately transferred to phosphate-buffered saline (PBS) and imaged using SHG (see description below). The second tendon was transferred to a solution of 1 μg/ml Alexa Fluor 555 labeled WGA (wheat germ agglutinin, Thermo Fisher Scientific, Waltham, MA, USA) in PBS. This sample was incubated for 1 h in a 37° C bath. WGA was used here as a general tissue marker and intended in this case to localize to glycoproteins and proteoglycans in non-collagenous/non-fibrillar matrix of the tendon. After incubation, tendon samples were thoroughly rinsed in a PBS bath at room temperature to remove excess WGA. Immediately prior to imaging, samples were bisected with a scalpel either longitudinally (in the first tendon) or transversely (in the second) in order to view subtendon structure in these two planes. The cut side faced upwards toward the microscope objective.

Tendons were imaged on an upright multiphoton microscope (TCS SP8 DIVE; Leica Microsystems, Wetzlar, Germany) using a water immersion objective (IRAPO L 25 × /1.00 W; Leica Microsystems) and a pulsed laser (InSight X3 Dual; Spectra-Physics, Santa Clara, CA, USA). Collagen was visualized by SHG imaging microscopy using the 920 nm laser line for excitation and a small detection window centered at 460 nm. The fluorescence of WGA-Alexa Fluor 555 was imaged in two-photon fluorescence excitation using the 1,045 nm laser line and a detection window from 577 to 633 nm. 3D imaging was performed by recording z-stacks (442.9 μm × 442.9 μm in x-y, between 80 and 225 μm z-range). In order to image over 2–4 mm in x- and y-direction, several z-stacks were recorded in tile scanning mode. Stitching and image processing was done in Leica LAS-X (Leica Microsystems).

### Finite Element Modeling of Achilles Subtendons

In this study, we used ten subject-specific FE models of the Achilles tendon generated from human cadavers and performed *in silico* experiments in various aspects of tendon fascicle twist angles. The finite element (FE) mesh of the human Achilles tendon was developed using the Visible Human dataset (Ackerman, 1998) using high order cubic Hermite elements that preserve both the continuity of nodal values and their first derivatives. As in our previous tendon studies (Shim et al., 2014, 2018; Hansen et al., 2017), we used Free Form Deformation (Fernandez et al., 2004, 2018) to morph the generic tendon mesh to match morphometrical features of individual participants whose tendons were scanned with ultrasound. Features included length, average cross-sectional area, anterior-posterior length, and medial-lateral length at 50% of the tendon length.

Fascicle twist was incorporated into the model with the structure-based material coordinate system using an FE field fitting procedure. First, the structure-based material coordinate system was rotated to reflect the degree of fascicle twist and then the initial FE reference material coordinate system was rotated using three sequential rotations based on Euler angles to align it with the prescribed fascicle twist. These Euler angles were then fitted as a finite element field (Mithraratne et al., 2010). We repeated this for four different twist angles−15, 30, 45, and 60°–for 10 subject-specific FE models of the tendon from our previous study (Shim et al., 2014). We then performed tendon rupture experiments with these models by performing uniaxial stretch and measuring von Mises stress until it reached the failure load (Wren et al., 2001). The predicted rupture loads for different twist angles were analyzed using one-way ANOVA to test for significant differences between twist angles (*p* < 0.05). Sensitivity analyses were performed to comparatively characterize the influence of the following factors on tendon strength: (1) fascicle twist angles, (2) cross sectional area (CSA), and (3) tendon stiffness. Parameters were varied according to means and standard deviations reported in the literature (van Gils et al., 1996; Wren et al., 2001, 2003).

Subtendon models were generated from the morphed subject-specific model, and based on the subtendon geometry described above. For computational simplification, we combined the medial and lateral gastrocnemius subtendons into a single “gastrocnemius subtendon,” creating a model consisting of a soleus subtendon and gastrocnemius subtendon. Note that while this model is appropriate for probing the sliding mechanics between the gastrocnemius and soleus subtendons, it should not be taken to imply non-independence of the two gastrocnemius subtendons. The element boundary between our subtendons was formed using the previous anatomical studies that showed subtendon boundaries in dissected human Achilles tendons (Szaro et al., 2009; Pekala et al., 2017).

The material model used here was based on a transversely isotropic hyperelastic material model that treats the tendon as a composite of collagen fascicles embedded in a matrix of ground substance (Weiss et al., 1996). Values for material property terms were taken as the average *in vivo* material properties estimated in our previous study (Hansen et al., 2017). Briefly, in that study we used 3D ultrasound *in vivo* to measure tendon deformation under maximum voluntary isometric contraction (MVIC). Using resting and 70% MVIC value, we found optimum parameters for the following three terms in the material model—the stiffness of ground substance, rate of collagen fiber loading, and Young's modulus of straightened fibers. Values used were the average from eight participants involved in the aforementioned study.

We modeled the contact condition between subtendons as frictionless contact. Differential muscle forces for soleus and gastrocnemius muscles were estimated by combining experimental data and musculoskeletal modeling that used a dynamic optimization algorithm by taking into account muscle-tendon dynamics of lower limb muscles (Swinnen et al., 2018) Briefly, four male participants (*n* = 4, age: 25.0 ± 2.4 years, height: 180.8 ± 3.0 cm, body mass: 69.7 ± 4.3 kg) performed rehabilitation exercises based on Alfredson's protocol (Alfredson et al., 1998) while we performed 3D motion capture (100 Hz; Vicon, Oxford Metrics, UK). Rehabilitation exercise protocols were ethically approved by the University Hospital Leuven Protocol S63532, and all participants provided informed written consent. The exercises included one-legged heel drop with and without bent knee, two-legged heel drop with and without extra weight (10 kg), toe- and heel walking and one-legged hopping. Ground reaction force during exercises were measured using a force plate embedded in the ground at a sampling frequency of 1,000 Hz. Each exercise was performed three times. We scaled a generic gait2392 model to subject-specific dimensions using OpenSim 3.3 (Opensim, Stanford, USA)(Delp et al., 2007). Joint angles were calculated using Kalman Smoothing and joint moments using an inverse dynamics approach. The muscle forces were then estimated using dynamic optimization with joint moments, muscle-tendon unit lengths and moment arms as inputs to solve the muscle redundancy problem by minimizing muscle activations squared (De Groote et al., 2016). For each exercise the peak soleus muscle force was found, and the soleus and gastrocnemius muscle forces at that point were applied to the subtendon sliding model as force boundary conditions where the bottom of the mesh was fixed, and the force was applied to the top surface of each subtendon as distributed forces. Two different contact conditions were used in our simulation—(1) frictionless contact to simulate subtendon sliding; (2) tied contact to simulate impaired subtendon sliding. We measured maximum displacement of each subtendon and maximum strain at the interface between subtendons. Note that differential motion between subtendons occurs even with a tied contact interface via tendon shearing.

The FE analysis was performed using CMISS (https://www.cmiss.org/), the computational framework developed as a part of the International Union of Physiological Society (IUPS) Physiome Project (Hunter et al., 2002; Hunter and Borg, 2003). The software is freely available for academic use (http://physiomeproject.org/software/opencmiss).

## Results

### Fascicle Twist and Sliding

**Figure 2:**
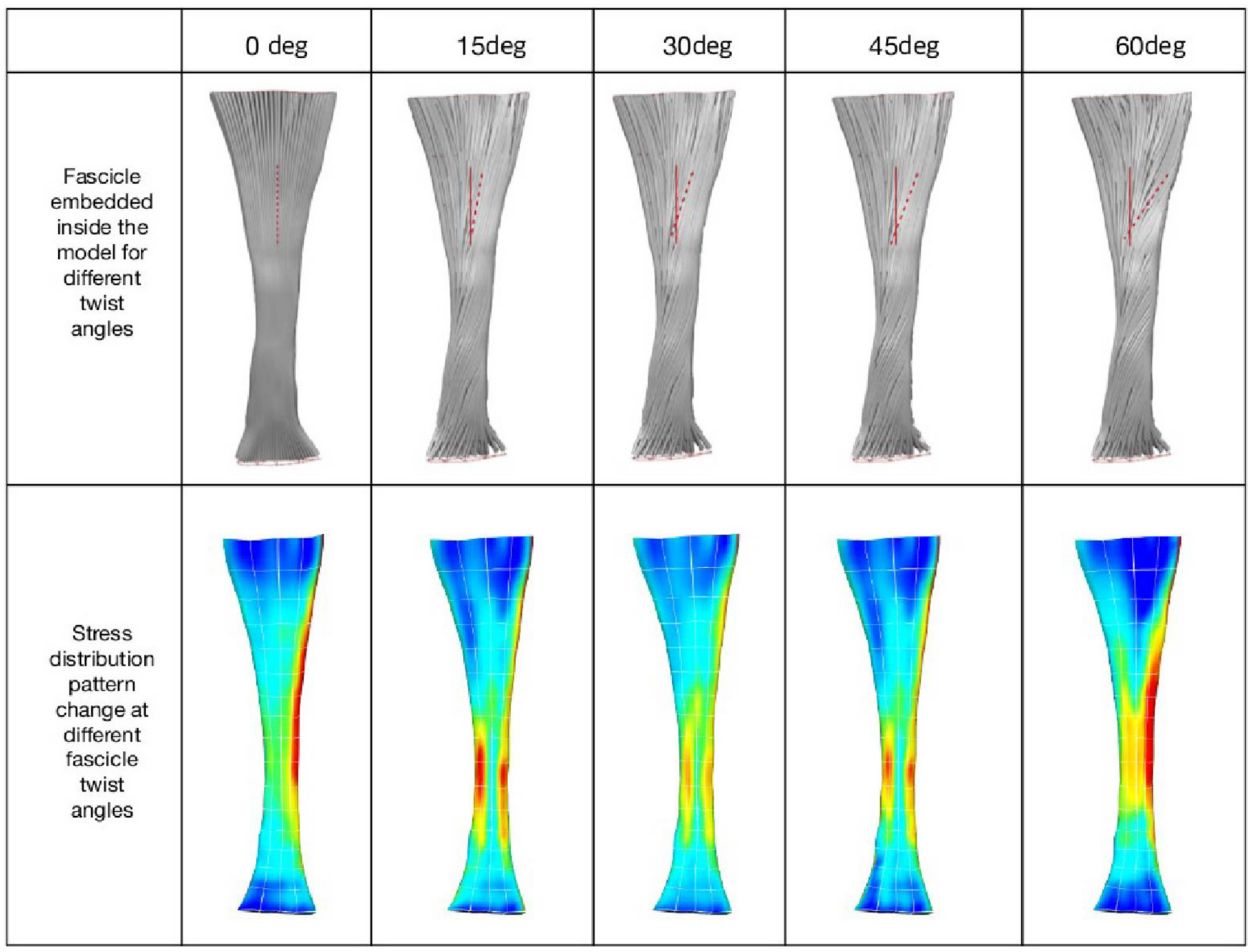
**(Top)** Fascicle twist implemented in our FE models. It is an anterior view with medial on the left and lateral on the right. **(Bottom)** von Mises stress distribution across five fascicle twist angles. Posterior view with medial on the right and lateral on the left side. Adapted from Shim et al. (2018).

When tendon fascicles were modeled using a continuum-based approach, we observed a minimization of peak stresses that occurs at the typical human fascicle twist angle, implying a functional role for the structure of fascicle twisting, which is to even out peak stress/strains within the tissue. As can be seen in Figure 2, stress distribution patterns varied with the different amount of fascicle twist angles. When no fascicle twists are present (0° twist), the stress concentration occurs on the medial side of the tendon. However, introduction of fascicle twist from 15 to 60° redistributes this stress concentration from the medial to lateral side. When the fascicle twist angle is around 30°, there occurs a nearly even distribution of the peak stresses on the medial and lateral sides, essentially relieving stress concentrations. Interestingly, this value of fascicle twist roughly coincides with the findings from a previous anatomical study by van Gils et al. (1996) who measured fascicle twist angles from 16 human cadaver specimens and found an average of 37°.

**Figure 3:**
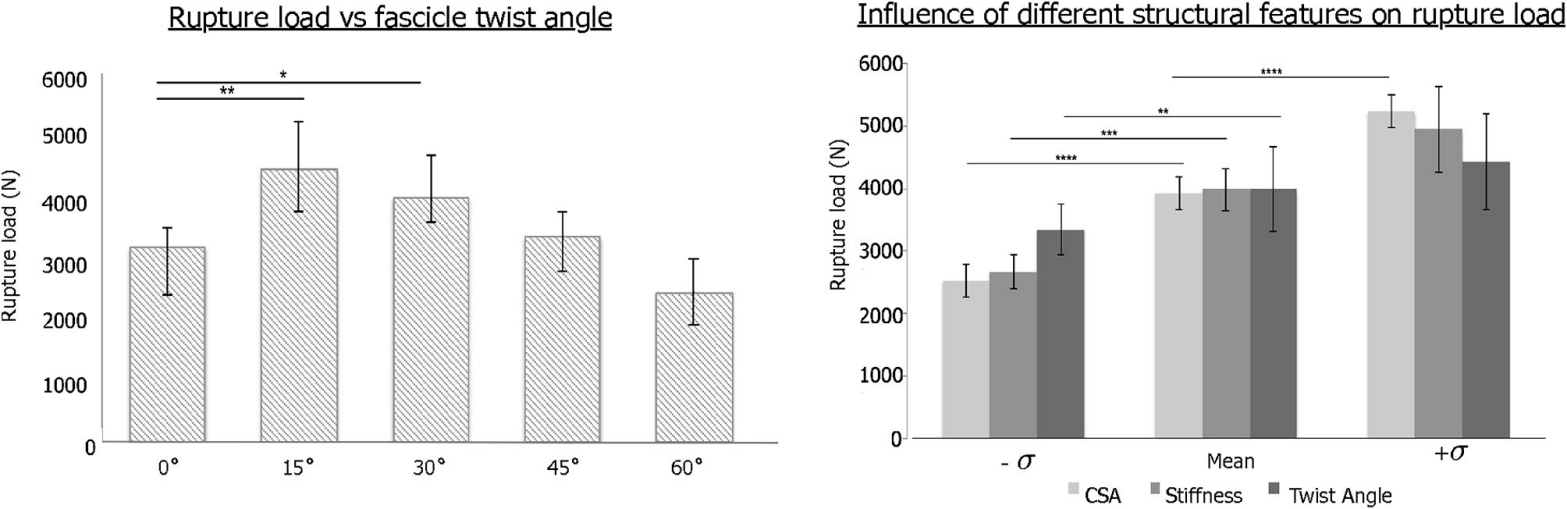
**(Left)** Tendon strength significantly improved by up to 40% when the fascicle twist angle is <30° (*n* = 10 with *p < 0.05, **p < 0.01, ***p < 0.001). **(Right)** Sensitivity analysis revealed that tissue strength improves with increases in CSA, stiffness and fascicle angle. However, Twist angle contributes less to the tissue strength than either stiffness or CSA (*n* = 10 with *p < 0.05, **p < 0.01, ***p < 0.001, and ****p < 0.0001) [error bars represent standard deviation]. Adapted from Shim et al. (2018).

The change in stress distribution pattern has a direct bearing on the tissue strength. When tendon rupture was simulated using the failure criteria based on a previous experimental study that reported tendon rupture load (Wren et al., 2001), we found that fascicle twist angles of 15 and 30° demonstrated improved tendon strength up to 40% compared to other twist angles (Figure 3). However, when the fascicle twist was put in perspective with respect to other structural features, such as cross sectional areas (CSA) and stiffness, we found the changes in CSA led to the significant changes in tissue strength while variations in the other structural features of fascicle twist and tissue stiffness did not lead to as great of changes as variations in CSA.

### Subtendon Structure and Twist

**Figure 4:**
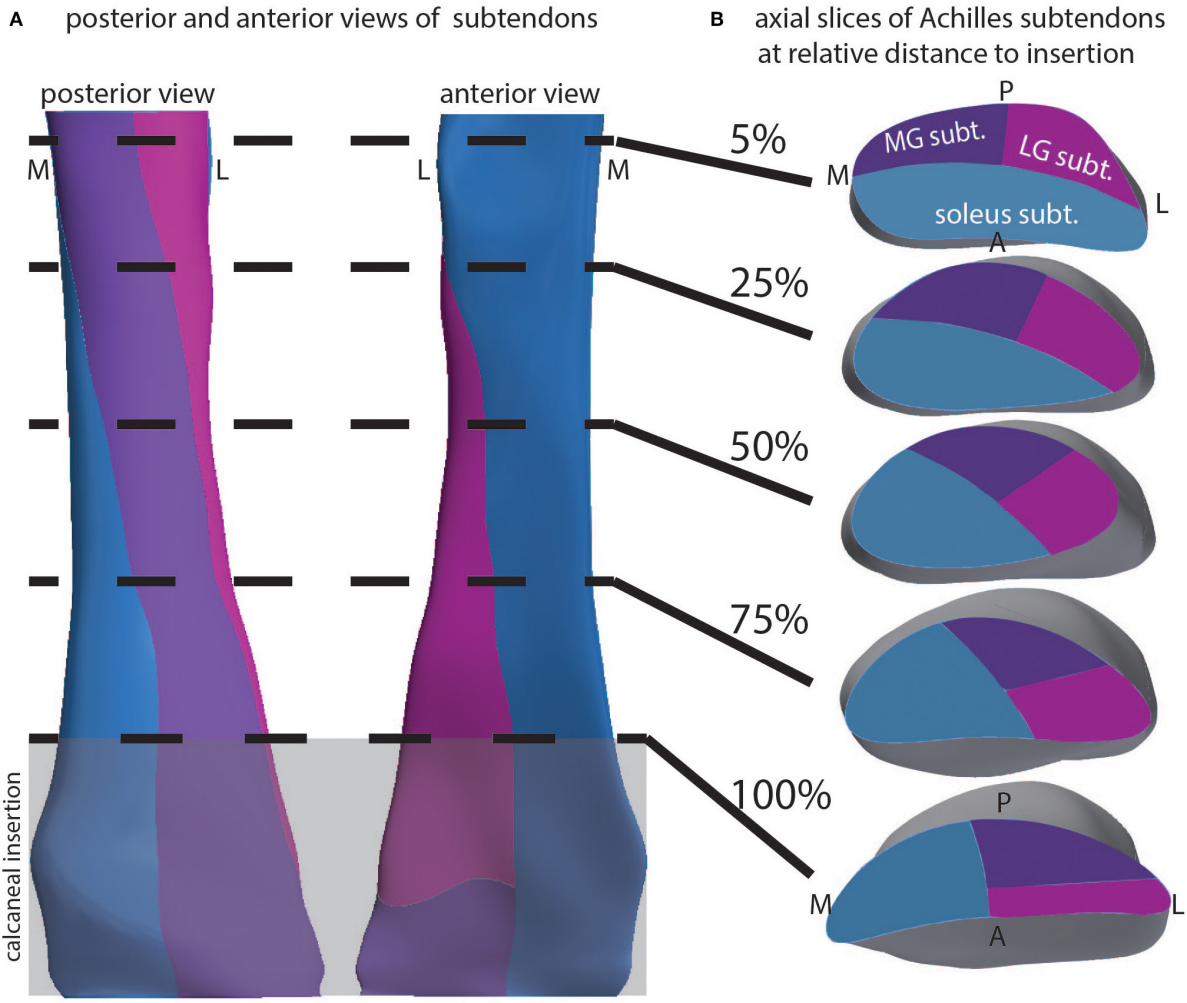
Depiction of the 3D structure of Achilles subtendons in whole tendon and cross-section. **(A)** Posterior and anterior views of the Achilles tendon show twisting subtendons. **(B)** Cross-sectional views at relative locations from the superior to inferior aspect of the free tendon, defined as the tendon distal to muscle and proximal to calcaneus. Calcaneal insertion is windowed in gray.

Our geometrical model was used to explore the 3D structure of the Achilles subtendons. The external geometry was determined from dual-echo UTE imaging of the Achilles. We used literature descriptions of the inferior aspect of the free tendon, as well as knowledge of the locations of the triceps surae muscles, to define the proximal and distal locations of the three subtendons. From this, interpolated cutting planes defined the 3D orientation of the subtendons (Figure 4A), which was consistent with anatomical literature. We probed the model with axial slices at relative locations through the length of the free tendon (Figure 4B). The LG subtendon originates on the posterior lateral aspect of the free tendon and inserts into the anterior lateral aspect. The MG subtendon originates on the medial posterior portion of the tendon, inserting onto the lateral posterior aspect, and represents the posterior aspect of the tendon through the center of the free tendon (see e.g., Figure 4B, 50% slice). The soleus subtendon originates on the anterior aspect of the free tendon and inserts on the medial aspect of the free tendon.

**Figure 5:**
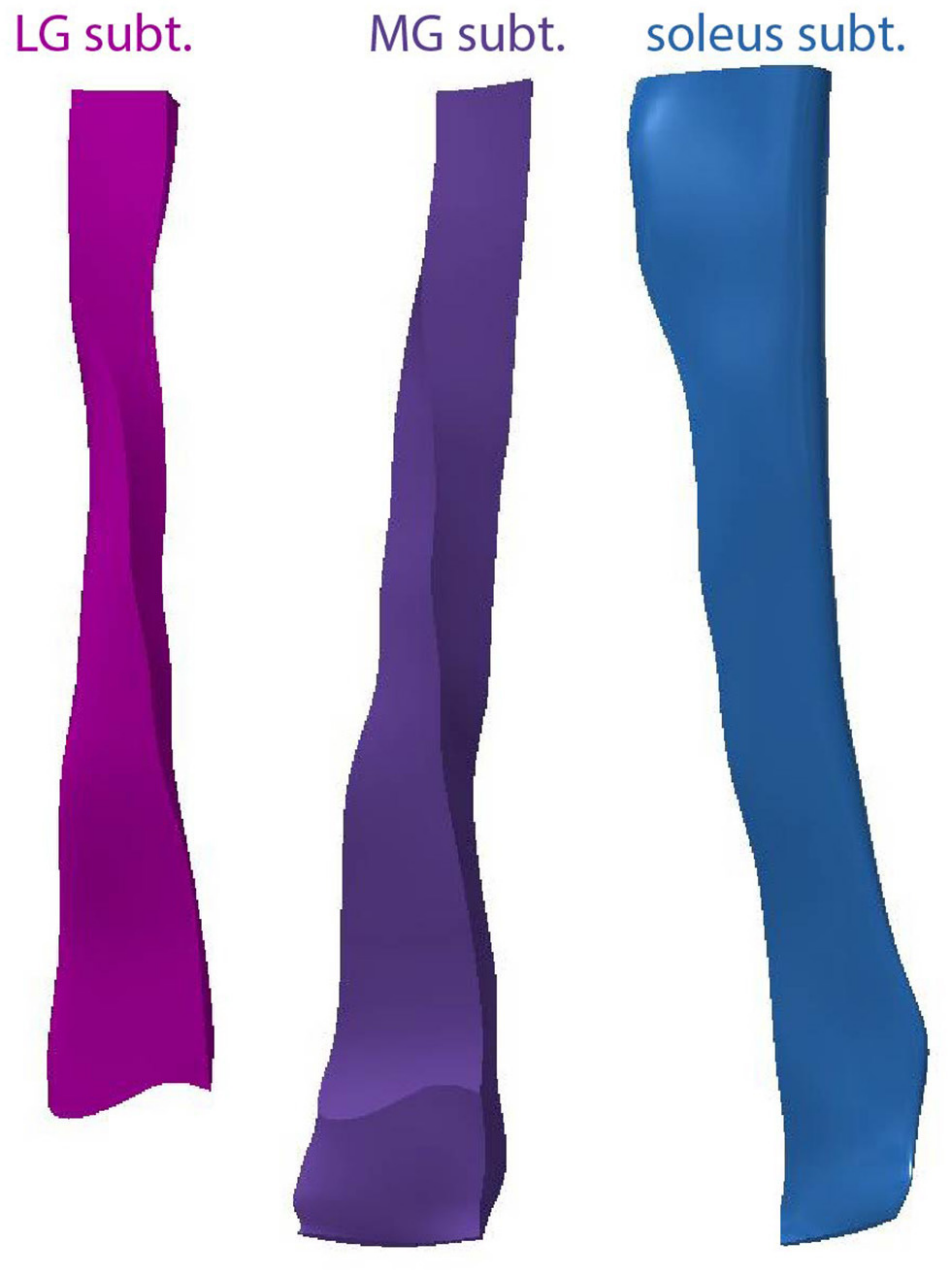
Anterior views of the three subtendons of the Achilles.

Using this model we separated the subtendons into individual structures (Figure 5). The soleus subtendon is the largest of the three and has the greatest area in the calcaneal region. The LG subtendon was shorter than the other two subtendons as it inserts into the calcaneus proximal to the MG subtendon. The LG subtendon was narrower in the distal free tendon as a consequence of this proximal insertion.

### Intersubtendon Matrix

**Figure 6:**
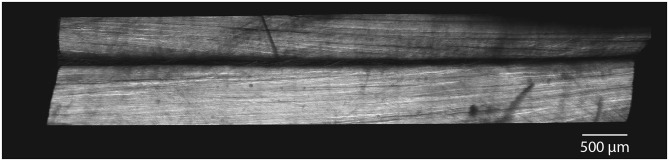
SHG imaging demonstrate distinct subtendons in rabbit Achilles tendons. In longitudinal section, images show two distinct subtendons with collagen fascicles present within each. A non-collagenous region exists between the subtendons with a width on the order of 30 μm.

Longitudinal images of rabbit Achilles tendons displayed subtendon structures within the tendon. Subtendons showed longitudinal collagen structures with evidence of collagen twisting within subtendons (Figure 6). Between subtendons was a non-collagenous region on the order of 30 μm in width (Figure 6).

**Figure 7:**
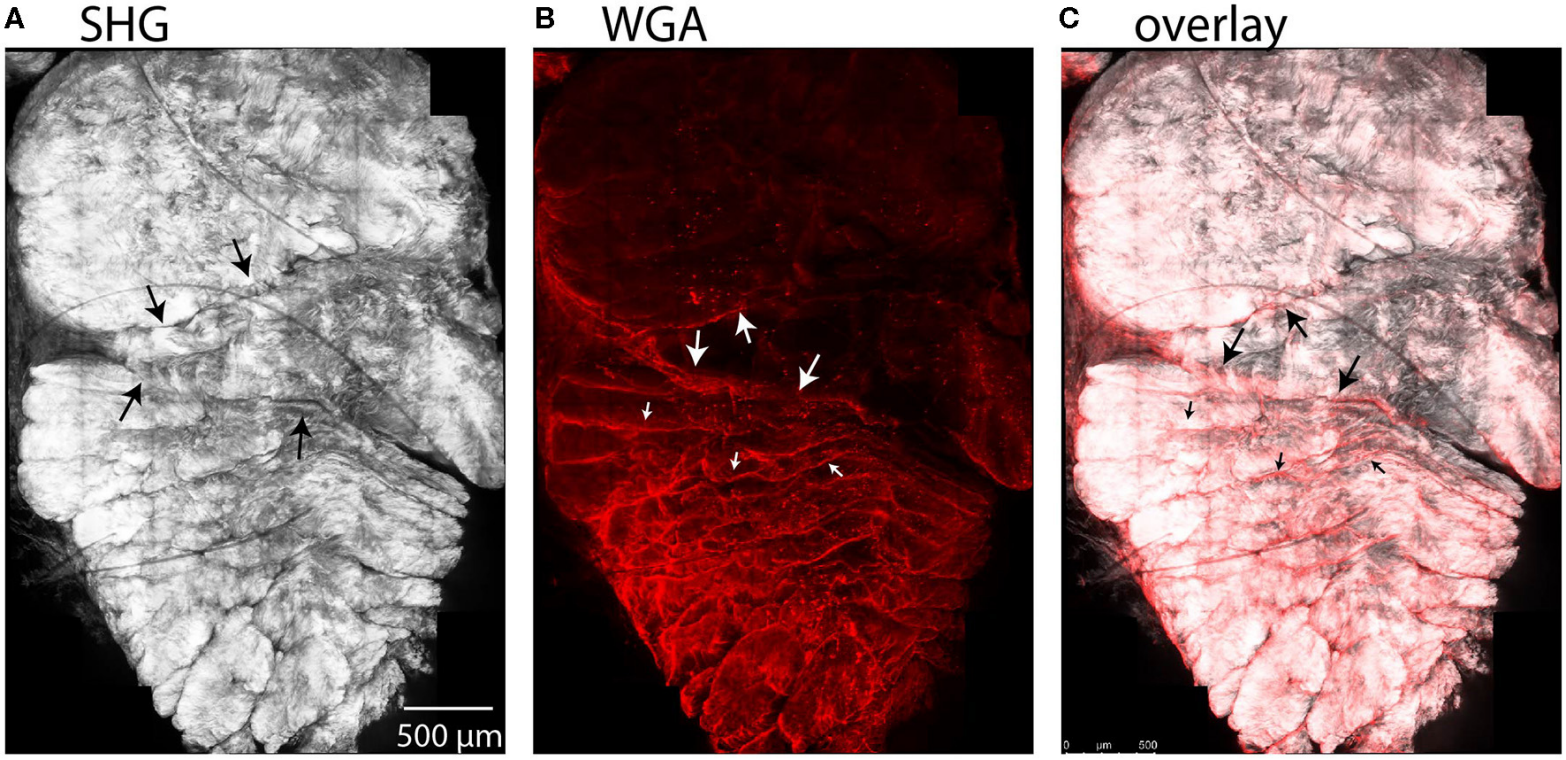
Maximum intensity projections in a cross-sectional view of rabbit tendon over 10 z-slices. **(A)** SHG imaging demonstrated fascicles within the subtendons of the rabbit Achilles tendon. **(B)** Two-photon fluorescence microscopy revealed that WGA is present in the inter-fascicular matrix (small arrows), as well as in the region between subtendons (large arrows), indicative of an inter-subtendon matrix. **(C)** Overlay of **(A)** and **(B)**.

We observed entry of WGA dye in the accessible region between the subtendons of the rabbit Achilles tendon in cross-sectional combined SHG and fluorescence images (Figure 7). Here, the SHG channel showed three subtendons in cross-section (Figure 7A). Fascicles are apparent within subtendons. The second channel shows fluorescently-labeled WGA that diffused into the inter-fascicular matrix as well as into the region between subtendons and bound there (Figure 7B), suggesting presence of an inter-subtendon matrix.

### Subtendon Sliding and Interaction With Muscles

The average material property values used in subtendon sliding analysis as well as the average muscle forces used are given in Tables 1, 2, respectively.

**Table 1:** Material properties used in the simulation.

Ground substance stiffness	40 Mpa
Collagen fiber uncrimping rate	13
Young's Modulus of straightened fiber	1390 Mpa

**Table 2:** Muscle forces used in the subtendon sliding simulation from four subjects for five different activities.

**Activity**	**Two legged heel drop**	**One legged heel drop**	**One legged heel drop with knee bent**	**Toe walking**	**Hopping**
Max Soleus muscle force (N)	680.6	1464.0	2371.2	1242.6	3777.4
Max Gastroc muscle force (N)	457.6	836.4	475.1	457.6	1816.8

**Figure 8:**
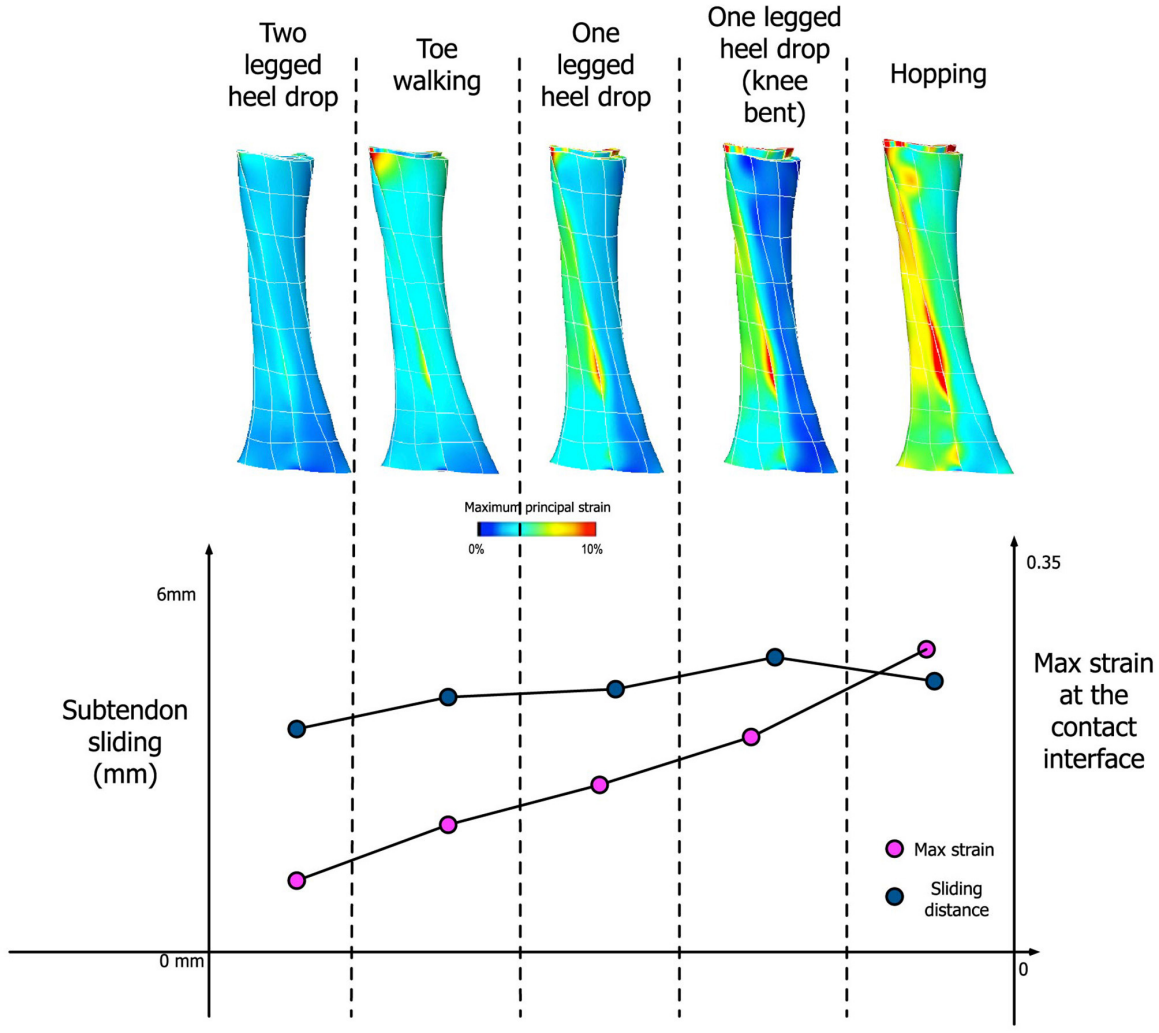
**(Top)** Achilles tendon strain distribution pattern from five different activities. **(Bottom)** Comparison between the amount of subtendon sliding and max strain developed at the contact interface for these activities.

Our simulation results show that the amount of sliding and maximum strain developed at the interface are dependent on the activity type, specifically the amount of muscle forces applied to each subtendon (Figure 8). Under the condition of frictionless subtendon sliding, the amount of muscle forces had influenced the maximum strain values in a directly proportional manner. Muscle forces also influenced the amount of subtendon sliding but the subtendon sliding was also dependent on the type of activity. As can be seen from Figure 8, the amount of subtendon sliding was higher for one legged heel drop exercise with knee bent than hopping despite having smaller muscle forces associated with it. However, the amount of strain developed at the interface was almost directly proportional to the amount of muscle force applied.

**Figure 9:**
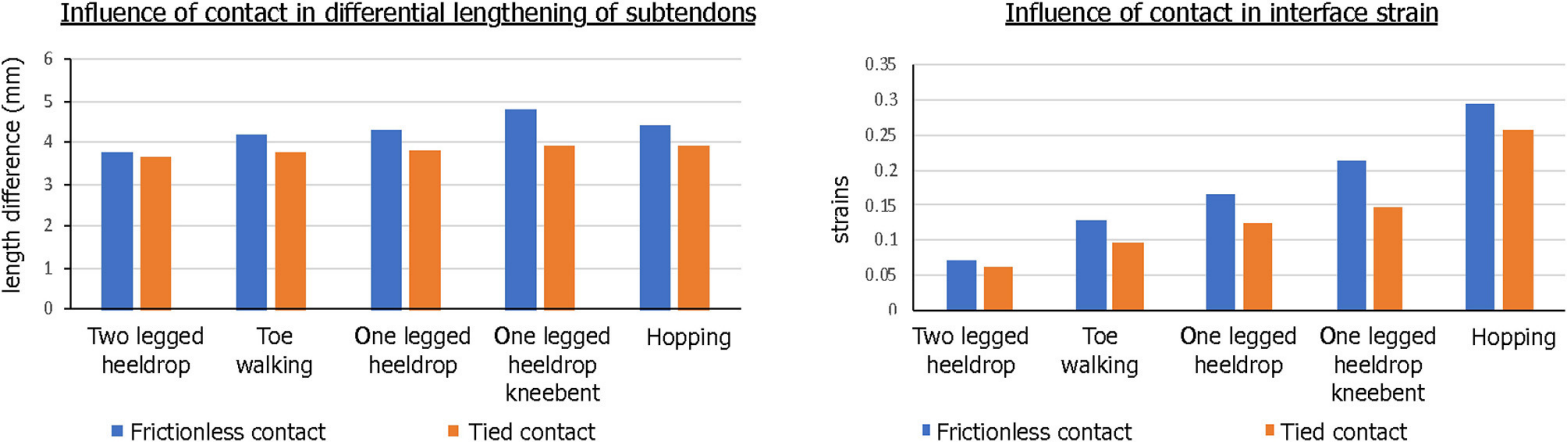
Influence of contact condition in differential motion of subtendons and interface strains. Both the differential lengthening distance and interface strain were lower for tied contact, simulating impaired sliding interface between subtendons.

The contact condition also played a role in the subtendon sliding and interface strains. When tied contact was used between the soleus and gastrocnemius subtendons in order to mimic the impaired sliding between the two subtendons, both sliding distance and interface strain developed were consistently lower and the differences were greater in the interface strains (Figure 9).

## Discussion

In this work, we used imaging and computational modeling to investigate the form and function of the subtendons of the Achilles tendon, to understand their behavior and how they may contribute to whole tendon function and disease. In this pursuit, we used non-Cartesian dual-echo MRI to acquire *in vivo* images of the Achilles tendon, 3D modeling to examine twisted subtendon geometry, SHG imaging to investigate the intersubtendon region in a rabbit model, and computational finite element modeling to explore the relationship between muscle forces and subtendon sliding during functional rehabilitation tasks. We further examined the functional role of fascicle twist using finite element modeling.

Ultrashort TE (UTE) MRI is an effective technique for imaging tendons in the human body and, in this study, was used to acquire 3D images of the Achilles tendon. While UTE uses an echo short enough to obtain signal from tendon and other collagenous tissues, similar signal intensity may concomitantly be obtained from surrounding tissues, especially muscle. To eliminate this unwanted signal and create high-signal-high-contrast images of the Achilles tendon, we performed dual echo UTE, where the second echo was acquired at a conventional short TE. Our final images were produced by subtracting the second echo from the first. Other imaging approaches have been used in the past to acquire tendon images, for instance ultrasound and magic angle imaging (Oatridge et al., 2001). Ultrasound is often limited to 2D planes, making it difficult to reconstruct the anatomy in 3D, although recent 3D ultrasound methods seem promising (Devaprakash et al., 2019). Magic angle imaging requires that the collagen fibers be aligned at 55° to the B0 field. This can prove cumbersome, particularly when the collagen does not have a linear trajectory within the tendon or when the tendon does not have a linear trajectory in the body, as is the case in the human Achilles.

Using the dual-echo UTE images, we performed image segmentation and developed 3D models of the Achilles tendon and its three subtendons. Subtendon geometry was created by subdividing the tendon into three parts at the superior aspect of the free tendon, and three parts at the superior aspect of the calcaneus. These locations were informed by literature descriptions of the subtendon locations (Sarrafian, 1993; Szaro et al., 2009). Using 3D modeling, the boundaries of the three subtendons were then interpolated from the superior to the inferior aspects, creating the 3D geometry of these structures. The created geometries display the internal torsion of Achilles substructures that have frequently been described and also illustrate the insertion location of each subtendon. Anatomical dissection studies have shown that collagen fibers, and the subtendons containing them, rotate about the central axis of the Achilles (White, 1943; van Gils et al., 1996; Edama et al., 2014, 2016). Furthermore, the specific orientations of the Achilles subtendons appear to be somewhat subject-specific (Edama et al., 2014; Pekala et al., 2017). Ideally, it would be beneficial to resolve the different subtendons of the Achilles tendon using *in vivo* imaging. This would allow for the creation of subject-specific models of healthy and tendinopathic individuals for simulation and investigation. Our present MRI methods did not allow us to resolve the Achilles tendon into unique subtendons—the subtendons we modeled were the result of assumptions based on literature information. Because we assume that each subtendon within an individual has similar material composition, imaging of subtendons requires that we resolve the matrix that separates the subtendons. Current resolution of our MRI methods is 800 μm. Given the apparent inter-subtendon matrix in rabbit, we propose that we would need 4–8 × greater resolution in order to resolve the intersubtendon matrix in humans.

We used SHG imaging to explore the microstructure of subtendons in a rabbit animal model. SHG is a non-linear optical effect that can occur on non-centrosymmetric molecular structures, such as collagen fibrils and bundles. The SHG channel revealed strong signal in the subtendons of the Achilles and in the fascicles within the subtendons, and revealed a 30 μm region between subtendons. Fluorescence imaging of the WGA stain, which we used as a more general tissue marker, revealed that WGA diffuses into the region between the subtendons, which we believe indicates the presence of an inter-subtendon matrix, since this region is without signal in SHG imaging. WGA interacts with GIcNAc or sialic acid residues and thereby visualizes the presence of such glyco-moieties in this region. Furthermore, within subtendons, WGA appeared to delineate fascicles; since an inter-fascicular matrix has previously been described (Thorpe et al., 2015, 2016), this suggests that there may be compositional similarity between the region between subtendons and the matrix between fascicles within the tendon. The presence of a matrix between subtendons would lend the structures to sliding, which has been suggested in previous computational models (Handsfield et al., 2017), experimental animal models (Finni et al., 2018), and imaging experiments (Slane and Thelen, 2014; Franz et al., 2015). It is unclear why there were regional differences in both surface binding and penetration of WGA. These may be related to differences in protein content between subtendons. Further work into the composition of this matrix is needed, particularly histological and protein analysis to probe more deeply the chemical nature of this region. Further work may help in determining the friction properties that may influence inter-subtendon sliding in the Achilles tendon. Caution is always warranted when applying results from animal models to understanding human anatomy. While the rabbit Achilles has often been used as an animal Achilles model and it displays subtendons within the Achilles complex, there are anatomical differences between human and rabbit Achilles which should be considered (Doherty et al., 2006).

In previous work, we used a continuum level finite element model to elucidate fascicle mechanics within the Achilles tendon. Our focus was on fascicle twist, especially its role in heterogeneous strain distribution within the tissue. We implemented a series of subject-specific Achilles tendon models to quantitatively describe the role that fascicle twist plays in tendon biomechanics. This was of particular interest as some previous studies identified heterogenous strain distribution within the tendon to be a precursor to overuse injuries and tendinopathy (Maganaris et al., 2004; Magnusson et al., 2008). Our results demonstrate that the feature of fascicle twist redistributes stress concentrations from one side of the tendon to both sides, improving overall tissue strength by up to 40%. Notably, the fascicle twist revealed as the optimum by this work is between 15 and 45°, where stress concentrations were diminished and the load required for rupture was high. This may be compared to the work of van Gils et al. (1996) who found a mean fascicle twist angle of 37° in humans.

In the present study, we further developed this continuum level FE model to incorporate separate subtendon models and their sliding effects under a number of different activities. Our modeling results confirmed that differential motions of subtendons occur for various different activities, such as heel drop exercises, toe walking or hopping. What is of particular interest is the relationship between muscle forces and the degree of sliding between subtendons. Generally, the amount of subtendon sliding increased when the amount of applied muscle force increased. However, sliding distance was also dependent on the type of activity. For example, hopping resulted in the highest muscle forces but that did not translate into the largest sliding distance between subtendons. Rather, heel drop with knee bent exercise led to the largest amount of subtendon sliding. This means that those activities that generate larger differential muscle forces between the soleus and gastrocnemius muscles are likely to generate more subtendon sliding, indicating the importance of the type of exercise performed when promoting subtendon sliding. An interesting question is whether inter-subject differences in tendons are such that different individuals may require markedly different exercises to achieve differential motion and sliding of their tendons. In this study, we only collected data on 4 males of relatively similar size and fitness. Expansion of this study in the future may suggest how specifically tailored an exercise need be in order to promote Achilles rehabilitation.

Our subtendon sliding model is consistent with previous findings indicating intratendinous sliding may be reduced in aging tendons (Thorpe et al., 2013; Slane and Thelen, 2015). We showed in simulation that when subtendon sliding was impaired—via a tied contact between subtendons—both interface strains and intratendinous sliding were significantly reduced, which we propose to be a mechanism of tendon degeneration in tendinopathic tendons (Wang, 2006). Moreover, considering the imbalance of muscle excitations and force between the three heads of the triceps surae, which varied greatly between people (Crouzier et al., 2018, 2019), subtendon sliding is likely to play a major role in designing rehabilitation exercise for tendinopathy. Our results showed that individual force-sharing strategies are influenced by the type of exercise and level of contraction (different muscle force magnitude and contribution between exercises) likely leading to different subtendon stress and strain. Such variations in individual responses may imply different structural adaptations when doing the same rehabilitation program for Achilles tendinopathy. This highlights a need for better individualized and personalized training contents using experimental data and simulations, as demonstrated by the existence of “sweet spot” or optimal tendon loading by Pizzolato et al. (2019). Beyond this, it is interesting to note the differences in tendon stresses across the rehabilitation exercises—for instance, toe walking is associated with lower overall stresses than calf-raises, which are performed early in the rehabilitation process. Simulation-informed rehabilitation may be useful in the future when tailoring prescriptions for Achilles tendinopathy.

Subtendon structure and mechanics have broad implications on Achilles tendinopathy and aging in humans. It is well-established that Achilles tendinopathy is more common in middle-aged or older individuals, as well as in distance runners (Kader et al., 2002; Slane and Thelen, 2015). Given the tendency for greater differential displacements within the Achilles tendon among younger humans (Slane and Thelen, 2015), we posit that age-related changes to the subtendons and the inter-subtendon matrix may be related to the presentation of Achilles tendinopathy. With this in mind, we hypothesize age-related changes that may contribute to Achilles tendinopathy independently or in conjunction: (1) focal adhesions that develop in the inter-subtendon matrix and limit subtendon sliding, (2) age-related stiffening of the intersubtendon matrix, (3) diminished strength in the gastrocnemius muscles that prevents the forces necessary to engage subtendon sliding. Previous research on the fascicular matrix and the interfascicular matrix (IFM) shows higher turnover of collagen and other proteins within the IFM and suggests that an age-related decline in this turnover may result in a buildup of proteins in the IFM that could diminish sliding (Thorpe et al., 2016). There may be parallels between mechanisms at the fascicle level and the subtendon level, and it is worth hypothesizing that a buildup of damaged proteins in the intersubtendon matrix may stiffen the intersubtendon matrix with aging, inhibiting sliding and contributing to tendinopathy. Prior work has established that the Achilles becomes more compliant with aging (Stenroth et al., 2012). It is unclear how this may relate to subtendon sliding, alterations of the inter-subtendon matrix, or the development of Achilles tendinopathy. Further research is needed to test these and related hypotheses and to probe the mechanisms that predispose young distance runners to tendinopathy. It may be that altered neuromuscular control with endurance training may affect muscle activation or protein turnover in a way that diminishes subtendon sliding, but this is hypothetical and warrants further investigation.

How might diminished inter-subtendon sliding promote Achilles tendinopathy? We believe this is a complex question where factors at multiple scales may influence the mechanics. We propose that inter-subtendon sliding may stimulate tenocytes in a way that promotes collagen turnover and healing of damaged tendon. A decline in sliding may cause a buildup of damaged collagen tissue which eventually becomes catastrophic and leads to overall swelling at the macroscale. We note that the finite element model presented here suggests a decline in inter-subtendon strain when sliding was prevented via a tied contact. This supports the idea that lack of sliding reduces strains, and may fall below a mechanical threshold needed to stimulate the tissue and promote health. Therapeutically, ice massage and eccentric exercises have been reported to alleviate Achilles tendinopathy (Kedia et al., 2014). It is possible that these methods work according to the same mechanism, by stimulating the tissue and promoting tenocyte activation. While we believe these are consistent ideas, at this time they remain hypothetical.

## Summary and Conclusions

The Achilles tendon is composed of three subtendons—each arising from different heads of the triceps surae muscle, twisting about one another in an internally rotated direction that is somewhat subject specific, and having the ability to differentially move and slide past one another in healthy subjects. Imaging and computational modeling were used here to probe the structure and behavior of the subtendons, and deepen our understanding of the structure-function relationships of features of the tendon. Dual-echo UTE MRI was used to acquire high contrast images of the Achilles tendon. Image-based finite element models suggest that the role of twist in subtendon structure is to reduce the tendon's rupture load and distribute stresses to avoid regional stress concentrations. Consistent with literature on observed twist angles in humans, computational modeling suggests an optimum twist between 15 and 45° optimizes rupture load and stress distributions. We found a 30 μm region between rabbit subtendons with SHG imaging, suggesting a non-collagenous inter-subtendon matrix which may facilitate subtendon sliding in the Achilles. Tendon rehabilitation exercises were studied and modeled to probe their effects on subtendon motion. We found that different exercises load the subtendons differently, where a one-legged knee-bent heel drop exercise maximizes subtendon sliding without maximizing subtendon strain. Inhibition of subtendon sliding reduced the interface strains and the differential displacement of subtendons in the Achilles. More work is needed in these exciting areas of research. Understanding the fundamental structure and mechanics of the Achilles and its subtendons is an important pursuit for identifying the pathogenesis of Achilles tendinopathy and for suggesting treatment and mitigation strategies in the future.

## Data Availability Statement

The raw data supporting the conclusions of this article will be made available by the authors, without undue reservation.

## Ethics Statement

The studies involving human participants were reviewed and approved by University Hospital Leuven S63532. The patients/participants provided their written informed consent to participate in this study. The animal study was reviewed and approved by Regierungspräsidium Freiburg, X-16/10R).

## Author Contributions

GH organized the team, wrote the bulk of the document, planned the section contributions, revised and prepared the document, and was the primary researcher on the MRI, geometrical modeling, and the SHG portion of the work. JG contributed to the development and data collection of the SHG portion of the work, and proofread, edited, and wrote sections of the document. JM conducted the technical aspects of the SHG work, oversaw data collection, and contributed sections to the document. ER-Z contributed to the experimental aspect of the SHG portion of this manuscript, oversaw sample preparation, interpreted results, proofread, and edited the document. EH and BV contributed technical aspects of the rehabilitation exercise portion of this work including data collection and analysis, contributed sections, proofread, and edited document. VS co-led the organization of this work, performed the continuum modeling portion of this work, wrote sections of the document, proofread, and edited.

## Conflict of Interest

The authors declare that the research was conducted in the absence of any commercial or financial relationships that could be construed as a potential conflict of interest.
